# Factors associated with suicidal ideation and suicidal attempts among adolescent students in Nepal: Findings from Global School-based Students Health Survey

**DOI:** 10.1371/journal.pone.0210383

**Published:** 2019-04-19

**Authors:** Achyut Raj Pandey, Bihungum Bista, Raja Ram Dhungana, Krishna Kumar Aryal, Binaya Chalise, Meghnath Dhimal

**Affiliations:** 1 Nepal Health Research Council, Ramshahpath, Kathmandu, Nepal; 2 College of Health and Biomedicine, Victoria University, Melbourne, Australia; Universita Cattolica del Sacro Cuore Sede di Roma, ITALY

## Abstract

**Introduction:**

Suicide has been recognized as a major public health problem with high burden in low and middle income countries. Suicide has long lasting psychological trauma on friends and relatives in addition to loss of economic productivity. Although the need of high quality evidence is essential for designing suicide prevention program, Nepal lacks reliable evidence from nationally representative data. This study aimed to estimate the prevalence of suicidal ideation and attempt among adolescent students and identify the factors associated with them.

**Materials and methods:**

Total of 6,531 students of grade 7 to 11 from 74 schools representing all three ecological belts and five development regions participated in this cross sectional study. To select the representative sample from study population, two stage cluster sampling method was used. Standardized self-administered questionnaire were completed by participants. Multivariable logistic regression was done to identify the factors associated with suicidal ideation and attempt.

**Results:**

Nearly 13.59% of the participants had considered suicide while 10.33% had attempted it. Food insecurity (OR = 2.32, CI = 1.62–3.32), anxiety (OR = 2.54, CI = 1.49–4.30), loneliness (OR = 2.51, CI = 1.44–4.36) and gender (OR = 1.39, CI = 1.03–1.89) were identified as risk factors of suicidal ideation. Anxiety (OR = 3.02, CI = 1.18–7.74), loneliness (OR = 2.19, CI = 1.28–3.73) truancy (OR = 1.99, CI = 1.40–2.82), cigarette use (OR = 3.13, CI = 1.36–7.23) and gender (OR = 1.60, CI = 1.07–2.39) were identified as risk factors of suicidal attempt. Having 3 or more close friends was found to have protective effect (OR = 0.35, CI = 0.16–0.75) against suicidal attempt.

**Conclusion:**

Study reveals relatively high prevalence of suicidal ideation and suicidal attempt among school-going adolescents in Nepal. Appropriate coping strategies for factors like anxiety, loneliness seem could be useful for preventing both suicidal ideation and attempt.

## Introduction

Suicide is one of the major causes of death and disability worldwide. A person commits suicide in every 40 seconds with almost 800,000 deaths annually [1]. The estimated age standardized rate for suicide was 10.67 per 100,000 population in 2015 [2]. Suicide is the second leading cause of death among 15–29-year-olds [3]. It largely affects low and middle-income countries accounting almost 78% of all suicide deaths globally [3]. Suicide rate in Nepal is 7.2 in general with 8.2 in male, and 6.3 in female per every 100,000 population [2]. Suicide in adolescence is often underreported with possible cause of death being classified as underdetermined or accident to protect the families from possible stigma associated with it [4].

Because of the sensitivity of the issue, suicide has received attention in global public health arena in recent years. In addition to loss of life and economic productivity for the society, there is long lasting psychological trauma on friends and relatives. Prevention of suicide is the best option given that most of suicide cases don’t get treatment nor they can be treated [5]. Target 3.2 of Mental Health Action Plan 2013–2020 has envisioned reducing the rate of suicide in WHO member countries by 10% by 2020 which requires clear understanding of the factors leading to suicide [6].

Although the epidemiological evidence are essential in designing interventions, suicidal behavior has a large number of underlying causes that are complex to understand and differ from one country to other thus making the preventive efforts more complex and diverse [7,8]. Both suicidal ideation and suicidal attempt share the common risk factors such as hopelessness, social isolation, anxiety, depression, impulsivity and substance abuse, among others. Several other adverse stimuli are also likely to be associated with the development of suicidal ideation and the progression from ideation to attempts [9]. Evidence suggests that adolescents of both genders who had suicide ideation and attempts are significantly more likely to commit suicide than those without such ideation and attempts [10].

Delivery of mental health services including treatment of suicidal attempts are limited especially in urban areas and is further constrained by limited human and financial resources. Government of Nepal spends less than 1% of total healthcare budget in mental health [11]. Thus, suicide prevention program are crucial in context of Nepal. Suicide prevention activities need to be tailored as per the context of the country and need deeper understanding on the determinants of the suicide. However, Nepal lacks reliable and representative data due to multiple reasons like poor registration system, mis-categorization of suicide cases by hospitals, and underreporting of suicide incidences in police data because of stigma attached with suicide [11]. Although there are some data from hospital based studies confined to specific setting and small scale cross sectional studies, Nepal lacks large scale nationwide studies to guide policy making process [12,13]. Identification of factors associated with suicidal ideation and attempt among adolescent students can be helpful in designing the appropriate interventions and reducing the burden of condition [14]. In this context, this study was designed to assess the determinants of suicidal ideation and attempts among adolescents of Nepal.

## Materials and methods

Data used in this study were obtained from Global School-based Students Health Survey (GSHS) 2015 which was a nationwide study using a globally standardized methodology. The research was approved by Ethical Review Board of Nepal Health Research Council. Prior to participation of student in survey, administrative permission from respective schools and written informed consent from student's parent and students were obtained ensuring voluntary participation, privacy and confidentiality. Students were also informed beforehand about the study explaining about the objective of the study, potential risk and benefits, confidentiality and privacy the information provided.

Two-stage cluster sampling was used in this study to select representative sample from study population that comprised of 7 to 11 class students. In the first stage of sampling strategy, 74 schools were selected based on probability proportional to school enrolment size from the list of 20,304 schools containing any of 7 to 11 class. In second stage, sampling frame comprising of the list a classrooms in each of selected school was prepared and intact classrooms were selected based on predetermined random numbers. Each student in the selected classroom were eligible to participate in the study. Probabilities of selection of each participants and non-response rate were adjusted applying appropriate weighting factor. Out of 74 schools selected for the study, 68 schools (92%) participated in the study. Four schools were closed even on the third visit, one school could not be reached because of disruption of road due to flood and one refused to participate.

All students in selected class were explained about the study and were provided with information paper about GSHS and a consent form to be signed by parents on the first day and were requested to share the information sheet with parents before obtaining consent from parents for participation in the study. The following day, questionnaire was administered among students who had consent from parents and were willing to participate in the study. From among 8,670 students selected for the study, 6,531 participated making response rate of 75%. The age of the participants ranged from 11–18 years. Of the total completed questionnaire received back from students, 6,529 questionnaire were useable.

Data were collected through self-administration of standardized Nepali version of the questionnaire. The GSHS questionnaire used for this study contained total of 91 questions with 58 core and 33 expanded questions covering demographics of the students, dietary behaviors, hygiene, violence and unintentional injury, tobacco use, mental health, alcohol and drug use, sexual behaviors and physical activity. Variables used in this study, the questions used and their coding schemes are presented in Table 1.

**Table 1:** Variables used in the study.

Variable	Survey question	Coding
**Gender**	What is your sex?	1 = Male, 2 = female
**Age**	How old are you?	1 = 12 years or younger, 2 = 13 years, 3 = 14 years, 4 = 15 years, 5 = 16 years or older
**Food insecurity**	During the past 30 days, how often did you go hungry because there was not enough food in your home?	1 = Most of times/always, 0 = Never/rarely/sometimes
**Anxiety**	During the past 12 months, how often have you been so worried about something that you could not sleep at night?	1 = Most of times/always, 0 = Never/rarely/sometimes
**Loneliness**	During the past 12 months, how often have you felt lonely?	1 = Most of times/always, 0 = Never/rarely/sometimes
**Close friends**	How many close friends do you have?	0 = 0 close friends, 1 = 1 close friends, 2 = 2 close friends, 3 = 3 or more close friends
**Truancy**	During the past 30 days, on how many days did you miss classes or school without permission?	0 = 0 to 2 times, 1 = 3 or more times
**Bullied**	During the past 30 days, on how many days were you bullied?	0 = 0 times, 1 = 1 or more times
**physically attacked**	During the past 12 months, how many times were you physically attacked?	0 = 0 times, 1 = 1 or more times
**Physical fighting**	During the past 12 months, how many times were you in a physical fight?	0 = 0 times, 1 = 1 or more times
**Current cigarette use**	During the past 30 days, on how many days did you smoke cigarettes?	0 = 0 times, 1 = 1 or more times
**Initiation of drug use**	How old were you when you first used drugs?	0 = I have never used drugs, 1 = any other response
**Really drunk**	During your life, how many times did you drink so much alcohol that you were really drunk?	0 = 0 times, 1 = 1 or more times
**Parents check homework**	During the past 30 days, how often did your parents or guardians check to see if your homework was done?	1 = Most of times/always, 0 = Never/rarely/sometimes
**Parents understand Problem**	During the past 30 days, how often did your parents or guardians understand your problems and worries?	1 = Most of times/always, 0 = Never/rarely/sometimes
**Parental monitoring**	During the past 30 days, how often did your parents or guardians really know what you were doing with your free time?	1 = Most of times/always, 0 = Never/rarely/sometimes
**Parental intrusion of privacy**	During the past 30 days, how often did your parents or guardians go through your things without your approval?	1 = Most of times/always, 0 = Never/rarely/sometimes
**Parental support score**	Parental support score was computed by adding four variables: parents check homework, parents understand problem, parental monitoring and parental intrusion of privacy. Reverse scoring was done on the variable parental intrusion of privacy.	Reported in score from 0 to 4
**Suicidal ideation**	During the past 12 months, did you ever seriously consider attempting suicide?	0 = No, 1 = Yes
**Attempted suicide**	During the past 12 months, how many times did you actually attempt suicide?	0 = No, 1 = Yes

Data analysis was performed using STATA software version 15.0 (Stata Corporation, College Station, TX, USA). For all analysis, complex survey analysis was carried out. Weighted percentages are reported in descriptive analysis. Chi-square test and odds ratio (OR) were used for bivariate analysis to assess association between suicidal ideation and attempt and independent variables. Multivariable analysis was used for evaluation of the effect of explanatory variables for suicide ideation and attempt in the past 12 months (binary dependent variables) after adjusting for probable confounding variables. The two-sided 95% confidence intervals are reported in the results. Variable parental intrusion of privacy was omitted from multivariable logistic regression model because of multicollinearity.

## Results

Around 4.57% of research participants had faced food insecurity. Similarly, 4.36% had anxiety and 6.27% had felt lonely. Almost two third (65.77%) of the research participants had at least 3 friends. Slightly more than half (50.72%) had experienced bullying in school, 40.09% had experienced physical attack and 39.15% had been involved in physical fighting. Similarly, 6.17% had been using tobacco products and 8.58% had already initiated drug use. Around 13.59% had considered suicide while 10.33% had attempted suicide. Around 23.72% of research participants were of age 16 years or older. (Table 2)

**Table 2:** Characteristics of research participants.

		Male		Female		Total
	N	% (95% CI)	n	% (95% CI)	n	% (95% CI)
**Parental support score**						
**0**	111	4.07 (2.62–6.29)	127	4.53 (2.40–8.37)	238	4.31 (2.54–7.23)
**1**	886	31.61(27.13–36.45)	829	28.29 (23.23–33.95)	1715	29.90 (25.72–34.45)
**2**	763	25.67 (22.69–28.90	858	24.33 (21.87–26.97)	1621	24.98 (22.64–27.48)
**3**	941	29.21 (26.5,32.07)	1066	29.17 (24.95–33.79)	2007	29.19 (26.06–32.53)
**4**	315	9.44 (7.39–11.98)	526	13.68 (11.22–16.58)	841	11.62 (9.64–13.93)
**Food insecurity**						
No	2855	95.18 (92.87–97.18)	3256	95.67 (91.47–97.85)	6111	95.43 (91.95–97.45)
Yes	137	4.82 (2.82–8.13)	126	4.33 (2.15–8.53)	263	4.57 (2.55–8.05
**Could not sleep**						
No	2869	95.34 (93.87–96.48)	3245	95.91 (94.24–97.11)	6114	95.63 (94.68–96.43)
Yes	133	4.66 (3.52–6.13)	134	4.09 (2.89–5.75)	267	4.376 (3.57–5.33)
**Felt lonely**						
No	2774	93.39 (92.02–94.54)	3131	94.04 (92.39–95.35)	5905	93.72 (92.55–94.72)
Yes	192	6.61 (5.46–7.98)	190	5.96 (4.65–7.61)	382	6.28 (5.28–7.45)
**Close friends**						
0	101	3.82 (2.66–5.47)	157	5.05 (3.66–6.93)	258	4.45 (3.22–6.12)
1	304	11.10 (9.12–13.43)	507	16.26 (14.09–18.69)	811	13.73 (11.89–15.81)
2	425	14.83 (12.38–17.67)	543	17.21 (15.24–19.39)	968	16.05 (14.39–17.86)
3 or more	2141	70.25 (65.78–74.35)	2138	61.48 (56.88–65.78)	4279	65.77 (61.91–69.43)
**Truancy**						
0–2 times	2693	90.01 (87.15–92.29)	3054	91.58 (88.58–93.84)	5747	90.81 (88.32–92.81)
3+ times	263	9.99 (7.71–12.85)	246	8.42 (6.16–11.42)	509	9.19 (7.19–11.68)
**Bullied**						
No	1334	44.33 (39.85–48.91)	1838	53.83 (50.02–57.60)	3172	49.28 (45.57–52.99)
Yes	1505	55.67 (51.09–60.15)	1427	46.17 (42.4–49.98)	2932	50.72 (47.01-54-43)
**physically attacked**						
No	1548	49.71 (44.93–54.5)	2090	59.88 (55.02–64.55)	3638	54.91 (50.54–59.19)
Yes	1434	50.29 (45.50–55.07)	1251	40.12 (43.45–44.98)	2685	45.09 (40.81–49.46)
**Physical fighting**						
No	1750	56.32 (52.58–59.99)	2239	65.15 (60.91–69.16)	3989	60.85 (57.72–63.89)
Yes	1260	43.68 (40.01–47.42)	1153	34.85 (30.84–39.09)	2413	39.15 (36.11–42.28)
**Current cigarette use**						
No	2703	91.06 (88.52–93.08)	3236	96.47 (94.6–97.7)	5939	93.83 (91.97–95.28)
Yes	251	8.94 (6.92–11.48)	91	3.54 (2.30–5.40)	342	6.17 (4.73–8.03)
**Initiation of drug use**						
No	2548	88.53 (85.84–90.77)	3093	94.13 (91.82–95.82)	5641	91.42 (89.36–93.11)
Yes	311	11.47 (9.23–14.16)	165	5.87 (4.18–8.18)	476	8.58 (6.89–10.64)
**Really drunk**						
No	2729	92.77 (90.62–94.46)	3240	97.21 (94.96–98.47)	5969	95.04 (93.12–96.48)
Yes	208	7.23 (5.54–9.38)	74	2.79 (1.53–5.04)	282	4.96 (3.55–6.88)
**Parents check homework**						
No	1485	52.93 (48.45–57.37)	1532	48.90 (43.41–54.41)	3017	50.87 (46.47–55.26)
Yes	1478	47.07 (42.63–51.55)	1803	51.10 (45.59–56.59)	3281	49.13 (44.74–53.53)
**Parents understand problem**						
No	1388	49.03 (44.49–53.6)	1397	45.41 (39.51–51.45)	2785	47.19 (42.48–51.96)
Yes	1566	50.97 (46.4–55.51)	1899	54.59 (48.55–60.49)	3465	52.81 (48.04–57.52)
**Parental monitoring**						
No	1530	53.94 (49.39–58.43)	1401	46.69 (40.97–52.5)	2931	50.26 (45.58–54.93)
Yes	1452	46.06 (42.57–50.61)	1932	53.31 (47.5–59.03)	3384	49.74 (45.07–54.42)
**Age of respondent**						
**12 years or under**	259	10.99 (8.79–13.66)	333	12.84 (9.51–17.12)	592	11.94 (9.43–15.01)
13	526	19 (16.83–21.37)	639	20.37 (17.8–23.2)	1165	19.70 (18.09–21.43)
14	667	23.32 (20.53–26.34)	812	25.49 (23.03–28.12)	1479	24.43 (22.04–26.99)
15	651	20.26 (18.04–22.68)	782	20.15 (17.66–22.9)	1433	20.20 (18.39–22.15)
16 years or older	900	26.44 (23.74–29.33)	831	21.14 (17.89–24.82)	1731	23.72 (21.03–26.64)
**Suicidal ideation**						
No	2611	86.74 (83.29–89.58)	2897	86.08 (83.02–88.67)	5508	86.41 (83.81–88.64)
Yes	343	13.26 (10.42–16.71)	438	13.92 (11.33–16.98)	781	13.59 (11.36–16.19)
**Suicidal attempt**						
No	2758	90.26 (87.7–92.34)	3061	89.10 (86.21–91.45)	5819	89.67 (87.28–91.65)
Yes	248	9.74 (7.66–12.3)	324	10.9 (8.55–13.79)	572	10.33 (8.35–12.72

Adolescents having food insecurity were at 2 folds (OR = 2.32, CI = 1.62–3.32) higher risk of having suicidal ideation compared to those who had food security. Similarly, adolescents having anxiety had higher odds (OR = 2.54, CI = 1.49–4.30) of having suicidal ideation compared to their counterparts. Children who felt lonely had higher odds (OR = 2.51, CI = 1.44–4.36) of having suicidal ideation compared to their counterparts. Similarly, children who had initiated drug use were almost 2 folds (OR = 1.60, CI = 1.14–2.23) more likely to have suicidal ideation. Females had higher odds (OR = 1.39, CI = 1.03, 1.89) of having suicidal ideation compared to males.

Other variables like having parental support, having 3 or more close friends, truancy, physical fighting, current cigarettes use, getting really drunk, having parents who understand problems and check their homework were not found to have statistically significant association with suicidal ideation. (Table 3)

**Table 3:** Factors associated with suicidal ideation.

Variable	Bivariate analysis		Multivariable analysis	
	Odds Ratio	p	Odds Ratio	P
**Parental support Score (Ref: 0)**				
1	0.81 (0.585–1.13)	0.21	0.96(0.42–2.22)	0.97
2	0.57 (0.39–0.83)	0.005	0.77(0.28–2.08)	0.60
3	0.41(0.27–0.63)	<0.001	0.77(0.21–0.89)	0.69
4	0.24 (0.14–0.41)	<0.001	0.48(0.11–2.173)	0.33
**Food insecurity** **(Ref: No)**				
Yes	2.34(1.75–3.14)	<0.001	2.32(1.62–3.32)	<0.001
**Anxiety (Ref: No)**				
Yes	4.24(2.6–6.91)	<0.001	2.54(1.49–4.30)	<0.001
**Loneliness** **(Ref: No)**				
Yes	3.25(2.16–4.89)	<0.001	2.51(1.44–4.36)	<0.001
**Close friends** **(Ref: 0)**				
1 friend	0.61(0.39–0.94)	0.03	1.05(0.56–1.97)	0.86
2 friend	0.55(0.38–0.8)	<0.001	0.97(0.49–1.91)	0.93
3+ friend	0.33(0.24–0.46)	<0.001	0.65(0.36–1.17)	0.14
**Truancy (Ref: No)**				
3+	2.07(1.41–3.05)	<0.001	1.36(0.86–2.16)	0.18
**Bullied (Ref: No)**				
Yes	1.62(1.16–2.26)	0.006	1.38(1.034–1.83)	0.28
**Physically attacked** **(Ref: No)**				
Yes	1.42(1.12–1.81)	<0.001	1.23(0.99–1.54)	0.07
**Physical fighting** **(Ref: No)**				
Yes	1.49(1.21–1.82)	<0.001	0.99(0.73–1.33)	0.95
**Current cigarettes use (Ref: No)**				
Yes	2.9(1.88–4.47)	<0.001	1.66(0.93–2.92)	0.08
**Initiation of drug use (Ref: No)**				
Yes	2.82(1.98–4.02)	<0.001	1.60(1.14–2.23)	0.007
** Really drunk (Ref: No)**				
Yes	2.58(1.78–3.73)	<0.001	0.94(0.49–1.80)	0.86
**Parent check homework (Ref: No)**				
Yes	0.64(0.49–0.85)	<0.001	1.01(0.62–1.66)	0.95
**Parent understand problem (Ref: No)**				
Yes	0.6(0.46–0.78)	<0.001	0.913(0.56–1.47)	0.69
**Parental monitoring** **(Ref: No)**				
Yes	0.57(0.44–0.72)	<0.001	0.88(0.61–1.27)	0.50
**Gender (Ref: Male)**				
Female	1.13(0.90–1.43)	0.29	1.39(1.03–1.89)	0.04

Those adolescents who had anxiety were almost 3 folds (OR = 3.02, CI = 1.18–7.74) more likely to attempt suicide. Those who felt lonely most of times were almost 2 folds (OR = 2.19, CI = 1.28–3.73) more likely to attempt suicide compared to their counterparts. Having 3 or more close friends had protective effect (OR = 0.35, CI = 0.16–0.75) against suicidal attempts although having one or two close friends did not differ significantly from those not having any close friends. Truancy increased the risk of suicidal attempt by almost 2 folds (OR = 1.99, CI = 1.40–2.82). Similarly, those currently using cigarettes were 3 folds more likely (OR = 3.13, CI = 1.36–7.23) to attempts suicide while girls were around 2 folds more likely (OR = 1.60, CI = 1.07–2.39) to attempt suicide compared to their counterparts.

Other variables like having parental support, having food insecurity, physical fighting, initiation of drug use, getting really drunk, having parents who check their homework and understand problems were not found to have any statistically significant association with suicidal attempt. (Table 4)

**Table 4:** Factors associated with suicidal attempt.

Variable	Bivariate		Multivariable analysis	
	Odds Ratio	p	Odds	p
**Parental support Score (Ref: 0)**				
1	0.53 (0.36–0.77)	0.002	0.97(0.33–2.84)	0.95
2	0.30 (0.18–0.50)	<0.001	0.64(0.21–2.01)	0.44
3	0.19 (0.12–0.32)	<0.001	0.87(0.23–3.28)	0.82
4	0.15 (0.08–0.29)	<0.001	0.83(0.15–4.45)	0.82
**Food insecurity** **(Ref: No)**				
Yes	2.18(1.31–3.64)	<0.001	1.84(0.73–4.66)	0.18
**Anxiety (Ref: No)**				
Yes	5.02(3.48–7.23)	<0.001	3.02(1.18–7.74)	0.02
**Loneliness (Ref: No)**				
Yes	3.63(2.70–4.88)	<0.001	2.19(1.28–3.73)	0.01
**Close friends (Ref: 0)**				
1 friend	0.57(0.3–1.07)	0.08	0.56(0.26–1.23)	0.14
2 friend	0.41(0.24–0.72)	<0.001	0.57(0.26–1.22)	0.14
3+ friend	0.23(0.11–0.47)	<0.001	0.35(0.16–0.75)	0.01
**Truancy (Ref: No)**				
**3+**	2.58(1.84–3.62)	<0.001	1.99(1.40–2.82)	<0.001
**Bullied (Ref: No)**				
Yes	2.86(2.12–3.86)	<0.001	2.42(1.70–3.45)	<0.001
**Physically attacked** **(Ref: No)**				
Yes	1.83(1.36–2.46)	<0.001	1.42(0.90–2.22)	0.13
**Physical fighting** **(Ref: No)**				
Yes	2.09(1.59–2.75)	<0.001	1.18(0.86–1.63)	0.29
**Current cigarettes use (Ref: No)**				
Yes	0.65(4.59–9.25)	<0.001	3. 13(1.36–7.23)	0.01
**Initiation of drug use (Ref: No)**				
Yes	0.60(3.61–7.2)	<0.001	0.92(0.42–2.03)	0.84
**Really Drunk (Ref: No)**				
Yes	6.66(4.66–9.53)	<0.001	1.73(0.79–3.79)	0.16
**Parents check homework (Ref: No)**				
Yes	0.54(0.38–0.77)	<0.001	0.95(0.60–1.48))	0.80
**Parents understand problem (Ref: No)**				
Yes	0.51(0.39–0.67)	<0.001	0.77(0.491–1.22)	0.26
**Parental monitoring (Ref: No)**				
Yes	0.48(0.34–0.67)	0.08	0.86(0.59–1.26)	0.42
**Gender (Ref: Male)**				
Female	1.06(0.79–1.42)	0.70	1.60(1.07–2.39)	0.02

## Discussion

This study identified high burden suicidal ideation and suicidal attempts among adolescent students in Nepal. The study has also disentangled the influence of gender, loneliness, having close friend, anxiety, getting drunk, substance abuse, physical fighting and parental support over suicidal ideation and suicidal attempts. This is the first nationally representative study that estimated suicidal ideation and suicidal attempts in Nepalese adolescent students. Moreover, GSHS is conducted in multiple countries among nationally representative samples using globally standardized methodology thus making data comparable from one country to another and provide important opportunity to rectify gap in data on suicide [15]. However, the original study was not designed to determine the factors associated with suicidal ideation and suicidal attempts. Therefore, the factors identified in the study might not fully explain the suicidal ideation and suicidal attempts in the study population because of lacking information on key explanatory variables such as socio-economic status and psychological comorbidities.

Around 13.59% of the research participants had suicidal ideation and 10.33% had attempted suicide in our study. One of the previous study done in four cities in China (Wuhan, Urumqi, Beijing, Hangzhou) had reported that 17.4% of research participants had suicidal ideation and 8.1% had attempted suicide [8]. However, the other study in rural parts of China had found that 19% of the participants had suicide ideation and 7% had attempted suicide attempts in the past year [16]. Prevalence of suicidal ideation was 4.9% in Bangladesh, 11.6% in Bhutan, 13.1% in Maldives, 9.4% in Myanmar, 5.4% in Indonesia, 9.4% in Srilanka, 12.5% in Thailand and 9.3% in Timor-Leste [2,17,18,19,20,21,22,23,24]. GSHS has revealed that suicidal attempt was 6.7% in Bangladesh, 11.3% in Bhutan, 12.7% in Maldives, 8.8% in Myanmar, 3.9% in Indonesia, 6.8% in Srilanka, 13.3% in Thailand and 9.5% in Timor Leste [17,18,19,20,21,22,23,24]. Suicidal ideation and attempt rate seem to differ across countries. Suicidal behavior has a large number of underlying causes. The factors that place individuals at risk for suicide are complex and interactive [8]. Furthermore, access to different means of committing suicide might have created these differences in suicidal ideation and suicidal attempts in different countries.

Our study revealed that girls are at higher risk of suicidal ideation and suicidal attempt compared to boys. Findings from other studies regarding gender differences in suicidal ideation and attempt are not consistent. Most studies previous studies have shown gender differences in suicidal ideation. However, findings differ on whether boys or girls are at higher risk of ideation. Studies conducted in Canada, Uganda have shown higher rate of suicidal ideation among boys while other studies in Malaysia, China and Guyana have shown higher rates among girls [8,25,26,27,28,29]. On the other side, studies conducted in Lebanon, Tanzania, and Thailand have not shown any statistically significant difference of suicidal ideation between girls and boys [15,30,31]. Inconsistencies in gender differences in suicidal ideation and attempt might be due to social and cultural context of the country that defines status of girls in society. In case of Nepal, being male dominated society, problems of girls might have received less attention that motivated them to consider suicide. Furthermore, in some of the above studies the suicidal ideation might have been underreported by girls thereby showing lower suicide rate [15].

Our study revealed that children who felt lonely most of time or always are more likely to have suicidal ideation and suicidal attempt compared to their counterparts. Findings on association of loneliness with suicidal attempt and ideation are largely consistent in most of previous studies. Previous studies done in Lebanon, Uganda, Tanzania, Sub-Saharan Africa have also revealed that children are more likely to have suicidal ideation when they have feeling of loneliness [15,26,30,32]. Feeling lonely could exacerbate the ill effect of other problems associated with suicide behavior as they find no one to share about the problem that substantially alleviate the agony. This is further supported by another findings of our study that having 3 or more close friends had protective effect against suicidal attempts although having one or two close friends did not differ significantly from those not having any close friends. However, it’s equally important to note that our study revealed no statistically significant association between having close friends and having suicidal ideation. One of the previous study from China had also revealed statistically significant association between having close friends and suicidal attempt and failed to demonstrate any association with suicidal ideation [33]. Another study in Guyana has revealed lower risk of having suicidal ideation if the research participants have close friends [29]. This reinforces the importance of social and peer support in the role of maintaining mental well-being.

Similarly, Children who had anxiety had higher odds of having suicidal ideation and suicidal attempt. Findings are consistent with most of other studies done in different countries. Higher odds of having suicidal ideation was reported in previous studies done in Uganda, Lebanon, Thailand and Republic of Benin when children were worried [15,26,31,34]. Findings are quite usual as adolescents often consider suicide as means to overcome anxiety or distress in life.

There was no significant association between truancy or missing school without permission and having suicidal ideation in our study. The children who missed school at least 3 times without any permission were found to have almost 2 folds higher risk of having suicidal attempt. Previous similar study done in Republic of Benin had not found any significant association of truancy with suicidal ideation and attempts [34]. However, the variable should be dealt in conjugation with other factors like feeling lonely, having close friends and being worried.

Cigarettes smoking seems to increase the risk of suicidal attempt by almost 3 folds in our study. Some of the previous studies have also demonstrated the association between suicidal behavior and smoking [35,36,37,38,39]. There have been some possible explanations for association between smoking and suicide. Suicide has been linked with depression as people might tend to self-medicate themselves with nicotine opting for cigarette smoking or smoking can be personality characteristics associated with low self-esteem [40,41]. Depression might have worked as residual confounder in this study distorting the association of smoking and suicide. One of the previous study also suggests that the association between suicidal behavior and smoking might be because of some unobserved background variables like life circumstances. In the particular study, the use of fixed-effects regression models controlling for unobserved confounding sources had substantially reduced the magnitude associations between suicidal behavior and smoking [42]. Given the lack of any direct plausible causative mechanism, the interpretation of linkage between smoking and suicide need some precautions [43].

There are some limitations in the study. The existing stigma surrounding suicidal ideation and attempts in culturally diversified Nepalese societies might have caused an underreporting of the conditions leading to social desirability bias. Study does not cover the adolescent who did not attend school. Furthermore, study also did not collect information on socioeconomic status, religious affiliation, social participation and psychological co-morbidities that could be important in characterizing suicidal ideation and attempt. Further research could be useful in this regard. Despite these limitations, this is the first study done in Nepal among large and nationwide representative sample intended to determine the risk factors of suicidal ideation and attempt. Since the study has used globally standardized methodology of GSHS, study findings are also comparable to other countries adopting same or similar methodology. The study comes out with nationwide estimate of suicidal ideation and attempt among school going adolescents. The study findings could be useful for policy makers in designing appropriate strategies for suicide prevention. Adopting appropriate preventive strategies could be very useful in context of Nepal considering the limited availability of treatment services in rural areas constrained with lack of financial and appropriate human resources for mental health services.

## Conclusions

Study reveals high suicide rate of suicidal ideation and attempt among Nepalese school going adolescents. Factors like food insecurity, anxiety, Loneliness and gender were found to be associated with suicidal ideation while anxiety, loneliness, truancy, cigarette use and gender were found to be associated with suicidal attempt.
